# Two Group A Streptococcal Peptide Pheromones Act through Opposing Rgg Regulators to Control Biofilm Development

**DOI:** 10.1371/journal.ppat.1002190

**Published:** 2011-08-04

**Authors:** Jennifer C. Chang, Breah LaSarre, Juan C. Jimenez, Chaitanya Aggarwal, Michael J. Federle

**Affiliations:** 1 Center for Pharmaceutical Biotechnology, Department of Medicinal Chemistry and Pharmacognosy, College of Pharmacy, University of Illinois at Chicago, Chicago, Illinois, United States of America; 2 Department of Microbiology and Immunology, College of Medicine, University of Illinois at Chicago, Chicago, Illinois, United States of America; Children's Hospital Boston, United States of America

## Abstract

*Streptococcus pyogenes* (Group A Streptococcus, GAS) is an important human commensal that occasionally causes localized infections and less frequently causes severe invasive disease with high mortality rates. How GAS regulates expression of factors used to colonize the host and avoid immune responses remains poorly understood. Intercellular communication is an important means by which bacteria coordinate gene expression to defend against host assaults and competing bacteria, yet no conserved cell-to-cell signaling system has been elucidated in GAS. Encoded within the GAS genome are four *rgg*-like genes, two of which (*rgg2* and *rgg3*) have no previously described function. We tested the hypothesis that *rgg2* or *rgg3* rely on extracellular peptides to control target-gene regulation. We found that Rgg2 and Rgg3 together tightly regulate two linked genes encoding new peptide pheromones. Rgg2 activates transcription of and is required for full induction of the pheromone genes, while Rgg3 plays an antagonistic role and represses pheromone expression. The active pheromone signals, termed SHP2 and SHP3, are short and hydrophobic (DI[I/L]IIVGG), and, though highly similar in sequence, their ability to disrupt Rgg3-DNA complexes were observed to be different, indicating that specificity and differential activation of promoters are characteristics of the Rgg2/3 regulatory circuit. SHP-pheromone signaling requires an intact oligopeptide permease (*opp*) and a metalloprotease (*eep*), supporting the model that pro-peptides are secreted, processed to the mature form, and subsequently imported to the cytoplasm to interact directly with the Rgg receptors. At least one consequence of pheromone stimulation of the Rgg2/3 pathway is increased biogenesis of biofilms, which counteracts negative regulation of biofilms by RopB (Rgg1). These data provide the first demonstration that Rgg-dependent quorum sensing functions in GAS and substantiate the role that Rggs play as peptide receptors across the *Firmicute* phylum.

## Author Summary

Group A Streptococcus (GAS, *Streptococcus pyogenes*) is a pathogen responsible for a wide variety of diseases including pharyngitis and toxic shock, but is also commonly carried in the human population asymptomatically. What causes the switch between carrier and pathogenic states remains poorly understood. Cell-to-cell signaling in bacteria, also known as quorum sensing, is an important mechanism for virulence gene regulation in many pathogens, yet no conserved quorum sensing pathway has been elucidated in GAS. Here we identify and characterize a new GAS quorum-sensing circuit that consists of two regulators that respond to two secreted peptide signals. The regulators are members of a class of generally poorly studied proteins (known as Rgg) found throughout Gram-positive bacteria. The mature peptide signals, here termed pheromones for their ability to elicit responses in members of the population, enhance the production of biofilms, which have been associated with nasopharyngeal colonization, antibiotic-treatment failures, and necrotic infections. We found that exogenous addition of synthetically-produced pheromone also induced biofilm growth, demonstrating that GAS behavior can be manipulated by artificial signals and offering the possibility for alternative therapeutic treatments.

## Introduction

Major efforts are underway to understand the composition, dynamics, and function of the human microbiome and how it affects the health of the host. Thousands of bacterial species colonize the skin, mucosal surfaces, and gastrointestinal and respiratory tracts, where intimate interactions occur between cells of the same and different species, and between host and microbe [1], [2], [3]. The idea that chemical languages foster microbial communities and coordinate genetic pathways has emerged in recent years and describes a process commonly referred to as quorum sensing. Scores of signaling molecules have now been demonstrated to affect various behavioral states by controlling genes involved in virulence, competence, sporulation, fratricide, biofilms, and other traits [4], [5], [6], [7], [8]. Though many chemical signals have been identified, the number of languages that have been elucidated is relatively low given the diversity of human microflora.

*Streptococcus pyogenes* (Group A Streptococcus, GAS) is an obligate human resident and a member of the human microbiome; however, its colonization of individuals is thought to be transient, and it is commonly carried asymptomatically [9], [10]. GAS resides primarily in the oropharynx and on the skin and is capable of localized, suppurative infections, such as pharyngitis and impetigo [11]. In rare instances it becomes invasive, causing severe, life-threatening disease such as necrotizing fasciitis, myonecrosis, and toxic shock [12]. However, GAS can be isolated from individuals who show no signs of illness and do not respond immunologically to GAS antigens [13]. Very little is understood regarding expression of virulence genes during carriage. A critical question challenging our understanding of streptococcal biology is how GAS moves from a carrier state to a pathogenic one, and vice versa.

To successfully survive in the host, factors that enable GAS to simultaneously compete with other bacteria for nutrients while defending itself from the immune system and other bacterial offenses are highly regulated. Master regulators identified in GAS, such as CovRS, CcpA, and CodY, provide responses to various stimuli, including general stress, immunological attack and nutrient availability [14], [15], [16], [17]. Yet many other recognized GAS regulatory proteins control gene expression in response to changing environmental signals by indirect methods or by unknown pathways and have been designated ‘stand-alone’ regulators because their cognate sensory partners or the effectors that control their activity remain unidentified [18]. Three prominent stand-alone regulators of GAS are Mga (controlling expression of M protein and C5a peptidase, among others), RofA-like proteins (RALPs, regulating host attachment factors, Streptolysin S, and other regulators), and Rgg/RopB (controlling a secreted cysteine protease, SpeB, and other strain-specific targets).

Recently, we and others provided evidence supporting the notion that Rgg-family transcription factors serve as cytoplasmic receptors for intercellular signaling peptides [19], [20], [21]. An established paradigm for intercellular communication among low-G+C Gram-positive bacteria relies on peptide signaling molecules. Typically, ribosome-dependent polypeptides are produced as inactive pro-peptides, secreted from the cell, and processed into active signaling molecules. Secretion may rely on the general secretory (Sec) system or utilize designated transporters of the ABC-type [22], [23], [24]. A variety of proteases in different species enable the maturation process, and in some instances the peptide receives additional covalent modifications [23], [25], [26], [27], [28], [29], [30], [31], [32]. Signaling peptides, commonly called autoinducers, pheromones, or quormones (for quorum-sensing hormones), are detected either extracellularly by transmembrane sensor kinases, or intracellularly by import to the cytoplasm where they engage proteins affiliated with a signal-transduction pathway or a transcription factor directly. For peptides that are transported into the cytoplasm, the oligopeptide permease (Opp) serves to import peptides with a range of sizes [33]. Peptide preference during import has been documented for some pheromones and is determined by the extracellular subunit of the permease that is homologous to OppA [34], [35]. To date, in the best-characterized systems, peptide pheromones destined for the cytoplasm subsequently interact with proteins of the RNPP (Rap/NprR/PlcR/PrgX) family that contain tetratricopeptide repeats (TPR) with which the pheromone interacts directly [36], [37].

Until recently, the identity of a widespread, conserved quorum sensing pathway remained elusive among pyogenic species of streptococci, including GAS. Reports on *S. thermophilus* and *S. mutans* (non-pyogenic streptococci) demonstrate that Rgg family members exhibit activities that depend on secreted peptide pheromones in ways that resemble quorum-sensing pathways of other Gram-positive bacteria. The Rgg family, named after the first example termed *r*egulator *g*ene of *g*lucosyltransferase in *Streptococcus gordonii* [38], is characterized by an N-terminal helix-turn-helix domain and C-terminal region of approximately 220 amino acids rich in predicted alpha-helices. Although primary amino acid sequence homology among Rgg proteins is disparate, secondary and tertiary structure prediction algorithms (e.g. PHYRE and I-TASSER [39], [40]) reveal high structural similarity to PlcR and PrgX, two transcription factors of the RNPP family. Genome sequencing efforts reveal Rgg proteins to be widespread among low-G+C Gram-positive bacteria (*Firmicutes*), with several paralogs within each species.

A newly-defined activity for Rgg proteins comes from experimental analysis of genes encoding Rgg members and adjacent open reading frames (ORFs) that encode short, secreted peptides. One example is ComR, which, together with the short peptide ComS, positively regulates competence development in *S. mutans* and *S. thermophilus*. ComS is secreted, processed into an active pheromone, and re-imported to the cytoplasm where it presumably interacts directly with ComR to induce transcription of the alternative sigma factor gene, *sigX* [19], [21]. Highly homologous orthologs of *comR*/*comS* are conserved in all pyogenic, bovis, and mutans species of streptococci, and their functionality is under vigorous investigation. However, there is no report that any Rgg protein functions as a quorum-sensing effector in any pyogenic streptococci. Though a non-Rgg type peptide quorum-sensing pathway regulating some aspects of virulence has been demonstrated in GAS [41], [42], components of this system are poorly conserved and are absent from most clinical isolates.

*S. pyogenes* has four identifiable *rgg*-like genes including ComR (*spy49_0032*) and RopB (*spy49_1691*). RopB is well studied as being required for transcriptional activation of the secreted cysteine protease SpeB, an important factor in disease development [43], [44], [45]. Though a cell-density-dependent factor has been proposed as being necessary to activate *speB*, no such factor has been identified [46]. The remaining two *rgg*-like genes (*spy49_0415* and *spy49_0449c*) have not been characterized. Rigorous analysis of unannotated, small ORFs throughout Gram-positive genomes revealed a class of genes termed *shp*, for short hydrophobic peptide, that are commonly located near *rgg* genes [47]. By this analysis, ORFs categorized as *shps* are found adjacent to both *spy49_0415* and *spy49_0449c* in every sequenced GAS genome.

Here we demonstrate that two peptide pheromones encoded by *shp* genes serve to induce expression of neighboring genetic loci and positively regulate their own transcription. Additionally, biofilm biogenesis is promoted by these pheromones. This activity is mediated by the opposing activities of two Rgg-like proteins, one that de-represses gene expression and biofilm phenotype and one that activates them, in response to the extracellular pheromones, and depends on a trans-membrane peptidase (*eep*) for processing, and the oligopeptide permease (*opp*) for importation. These results provide further evidence that the Rgg family of transcriptional regulators, widespread among Gram-positive bacteria, function as quorum-sensing effector proteins and comprise the first functional quorum-sensing pathway conserved in all Group A Streptococci.

## Results

### Rgg2 and Rgg3 exhibit antagonistic effects at target promoters

**Figure 1:**
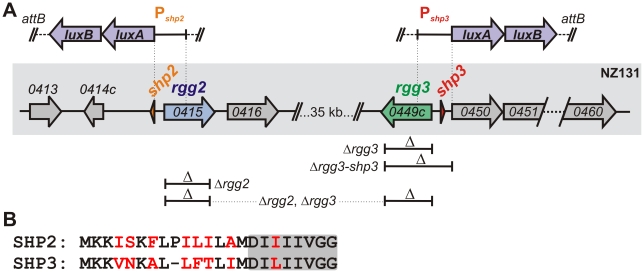
Rgg regulators in the *S. pyogenes* NZ131 genome. (**A**) The location of *rgg2* and *rgg3* and their neighboring genes is indicated by the gray box. Above the box, regions used to generate transcriptional reporters to *luxAB* are indicated by vertical dashed lines; these reporter constructs were inserted into the chromosome at the T12 bacteriophage *attB* site (*tmRNA*). Gene deletions are indicated below the box and correspond to genotypes listed in Table 1. (**B**) Alignment of pre-peptides SHP2 and SHP3 indicating identical amino acids in black font. The C-terminal eight residues are shaded.

The *Streptococcus pyogenes* NZ131 serotype M49 genome [48] contains four *rgg*-like genes that are conserved in all sequenced GAS genomes currently available; for simplicity, we have enumerated them *rgg1*-*rgg4*, ranked by their encoded amino acid sequence similarity to *ropB* (*rgg1*, *spy49_1691*): *rgg2* (*spy49_0415*, annotated as *mutR*); *rgg3* (*spy49_0449c*); and *rgg4* (*comR* [21], *spy49_0032*). The *rgg2* and *rgg3* genes encode proteins with a high level of similarity to one another (55% identical, 76% similar). Proximal to and encoded on opposite strands from both *rgg2* and *rgg3* genes (Figure 1A) are small, unannotated ORFs of 22 and 23 amino acids, respectively (NZ131 genome coordinates 412,519–412,584 bp and 449,894–449,962 bp). The predicted products of the small ORFs are also highly similar to one another (58% identical, 62% similar) (Figure 1B), and meet the criteria established by Ibrahim *et al*., [47] for designation as short hydrophobic peptides (SHPs); thus, we have named the genes *shp2* and *shp3* due to their proximity to *rgg2* and *rgg3*, respectively.

To test the central hypothesis that Rgg2 and Rgg3 function as quorum-sensing mediators to control gene expression in response to intercellular signals, we constructed mutant strains containing deletions of *rgg2* or *rgg3*, and a strain combining both deletions, in the chromosome of NZ131 (Table 1). To monitor effects of these mutations, we built transcriptional reporters to sites that we predicted might be targets of Rgg regulation. Many transcriptional regulators are located adjacent to the genes they regulate, and quorum-sensing regulatory circuits often contain positive feedback loops that rapidly induce the genes producing signaling molecules; therefore, DNA segments likely to encompass the promoters of the *shp2* and *shp3* genes (P*_shp2_* and P*_shp3_*) were selected for fusion to the bacterial luciferase genes, *luxAB*. The P*_shp2_* reporter contains the sequence directly upstream of *shp2*, while the P*_shp3_* reporter includes sequences that span *shp3* and the region immediately upstream of an apparent operon containing ten genes of unknown function that were found to be responsive to MtsR as described by Toukoki et al. [49] (Figure 1A). Transcriptional reporters were integrated into the chromosomes of wild type and mutant GAS strains in single copy at a phage attachment site (*attB*, [50]), which is distant from *rgg* and *shp* genes.

**Table 1:** Strains used in this study.

Strain	Description
NZ131	M49 serotype isolated from a case of acute post-streptococcal glomerulonephritis [48], [94]
BNL145	NZ131 Δ*rgg2*Δ*rgg3*::*cat*; Cm^R^
BNL146	NZ131 Δ*ropB*; unmarked
BNL147	NZ131 Δ*ropB*Δ*rgg2*; unmarked
BNL148	NZ131 with integrated pSar56 P*_shp2_* reporter; Erm^R^
BNL149	JCC131 with integrated pSar56 P*_shp2_* reporter; Cm^R^, Erm^R^
BNL150	JCC132 with integrated pSar56 P*_shp2_* reporter; Cm^R^, Erm^R^
BNL152	JCC137 with integrated pSar56 P*_shp2_* reporter; Erm^R^
BNL153	BNL145 with integrated pSar56 P*_shp2_* reporter; Cm^R^, Erm^R^
BNL165	NZ131 Δ*ropB*Δ*rgg3*::*cat*; Cm^R^
JCC131	NZ131 Δ*rgg3*::*cat*; Cm^R^
JCC132	NZ131 Δ*rgg3-shp3*::*cat*; Cm^R^
JCC133	NZ131 Δ*eep*; unmarked
JCC135	NZ131 Δ*oppD*; unmarked
JCC137	NZ131 Δ*rgg2*; unmarked
JCC140	NZ131 Δ*eep*Δ*rgg3*::*cat*; Cm^R^
JCC157	NZ131 with integrated pJC187 P*_shp3_* reporter; Erm^R^
JCC158	JCC131 with integrated pJC187 P*_shp3_* reporter; Cm^R^, Erm^R^
JCC159	JCC132 with integrated pJC187 P*_shp3_* reporter; Cm^R^, Erm^R^
JCC160	JCC133 with integrated pJC187 P*_shp3_* reporter; Erm^R^
JCC161	JCC140 with integrated pJC187 P*_shp3_* reporter; Cm^R^, Erm^R^
JCC163	JCC135 with integrated pJC187 P*_shp3_* reporter; Erm^R^
JCC166	JCC137 with integrated pJC187 P*_shp3_* reporter; Erm^R^
JCC167	BNL145 with integrated pJC187 P*_shp3_* reporter; Cm^R^, Erm^R^
JCC168	JCC132 with integrated pJC205 mutated P*_shp3_* reporter; Cm^R^, Erm^R^
JCC174	NZ131 with integrated pJC205 mutated P*_shp3_* reporter; Erm^R^
JCC175	JCC137 with integrated pJC205 mutated P*_shp3_* reporter; Erm^R^

Cm, chloramphenicol; Erm, erythromycin.

**Figure 2:**
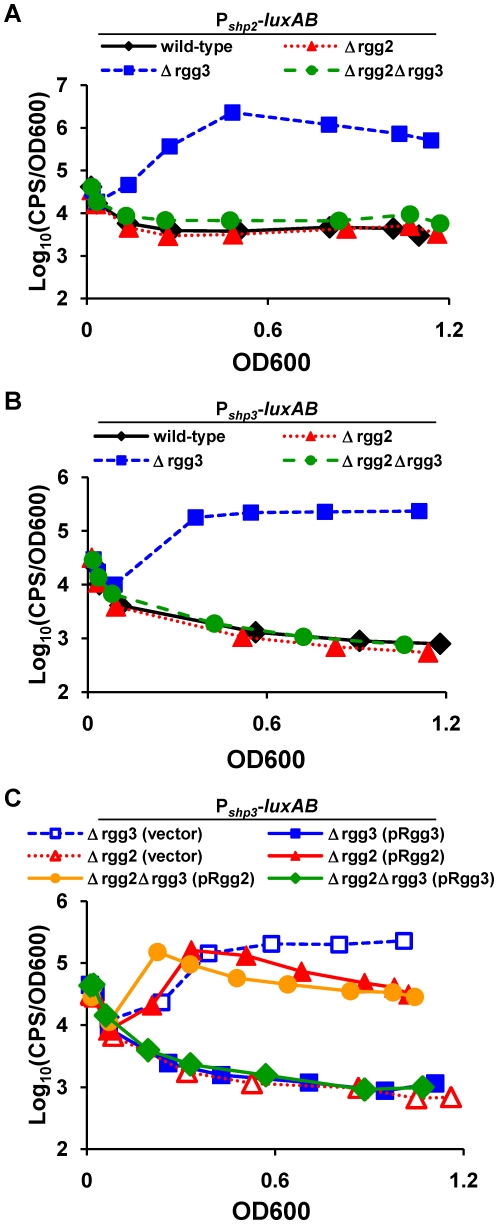
Gene regulation mediated by Rgg proteins. Overnight GAS cultures were diluted into CDM, and culture growth and reporter expression were measured over time. Data shown are representative of at least three independent experiments. Luciferase expression from (**A**) P*_shp2_* or (**B**) P*_shp3_* reporters integrated into wild-type (BNL148 and JCC157), Δ*rgg2* (BNL152 and JCC166), Δ*rgg3* (BNL149 and JCC158) and Δ*rgg2*Δ*rgg3* (BNL153 and JCC167) strains indicate activator and repressor functions for Rgg2 and Rgg3, respectively. (**C**) Complementation of selected strains with *rgg* genes driven by their native promoters confirms the antagonistic effects of Rgg2 and Rgg3 on *shp3* expression. Empty vector (pLZ12-Sp) was included as a control.

When luciferase activity of P*_shp2_* and P*_shp3_* reporters was monitored in wild-type (BNL148 and JCC157) or Δ*rgg2* (BNL152 and JCC166) strains during growth in an improved chemically defined medium (CDM; [51]; see Methods and Protocol S1), no detectable activity was observed (Figure 2A and 2B). However, P*_shp2_* and P*_shp3_* reporters were induced approximately 600- and 130-fold over background levels, respectively, as cells entered logarithmic phase and remained high until stationary phase in the Δ*rgg3* strain (BNL149 and JCC158), indicating that Rgg3 serves to repress each promoter. Examination of the double Δ*rgg2*Δ*rgg3* mutant strains (BNL153 and JCC167) revealed no luciferase induction from either promoter, signifying that the Δ*rgg2* mutation overrides effects of Δ*rgg3*. One possible scenario explaining this result is that expression of *shp* promoters requires Rgg2, and that Rgg3 blocks Rgg2-dependent activation.

Complementation experiments were also performed in which *rgg2* or *rgg3* was reintroduced to mutant strains on a multi-copy plasmid, and the effect on luciferase activity was observed. In the Δ*rgg3* strain (JCC158), expression of *rgg3* from pRgg3 returned luciferase activity to baseline levels (Figure 2C), confirming the role of Rgg3 as a repressor; unsurprisingly, pRgg3 had no additional effect in the double Δ*rgg2*Δ*rgg3* mutant (JCC167), which exhibited little reporter activity. In contrast, expression of *rgg2* from pRgg2 in the double mutant led to partial restoration of light production, consistent with its possible function as an activator at P*_shp3_*. Similar results were observed for P*_shp2_* reporter strains (data not shown). Interestingly, expression of *rgg2* in the Δ*rgg2* single mutant strain (JCC166) resulted in increased P*_shp3_* reporter activity, indicating that activation of *shp* promoters may be affected by the concentration of this regulator within the cell.

### Inducing activity is contained within the C-terminus of SHPs

**Figure 3:**
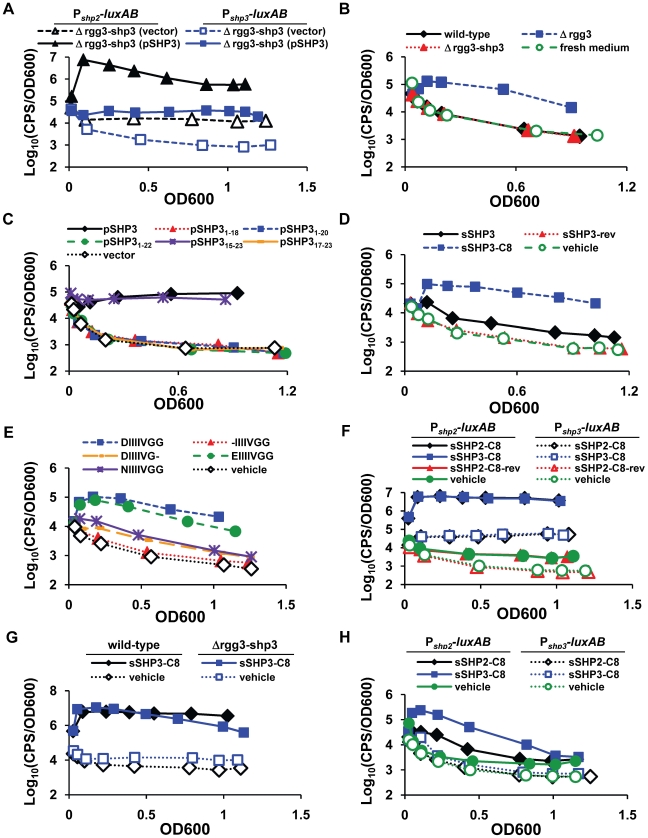
SHP stimulation of luciferase production. For panels A and C, overnight GAS cultures were diluted into CDM and followed for growth and light production over time. For panels B and D-H, reporter strains were grown to log-phase (OD600 between 0.3 and 0.5) and then diluted 13-fold into conditioned (panel B) or fresh CDM containing 50 nM of the indicated peptide (panels D-H). For all synthetic peptide experiments, DMSO was included as a vehicle control. Data shown are representative of at least three independent experiments. (**A**) Expression of *shp3* under its own promoter from a multi-copy plasmid (pSHP3) induces luciferase expression at both P*_shp2_* (triangles) and P*_shp3_* (squares); pLZ12-Sp is the vector-only control. (**B**) Luciferase-inducing activity is present in a cell-free culture supernatant prepared from mid-log phase Δ*rgg3* donor (JCC131) but not in wild-type (NZ131) or Δ*rgg3-shp3* (JCC132)-conditioned supernatants; Δ*rgg3-shp3* (JCC159) was used as the reporter strain, and fresh medium was included as a control. (**C**) Luciferase activity of a Δ*rgg3-shp3* strain (JCC168) expressing truncated versions of *shp3* indicates the importance of the C-terminus. All truncation plasmids were derived from pSHP3; pSHP3_17-23_ includes a methionine to initiate translation. (**D**) Synthetic full-length (sSHP3) and C-terminal eight amino acids (sSHP3-C8), but not reverse peptide (sSHP3-rev), induce luciferase activity in a Δ*rgg3-shp3* reporter strain (JCC168). (**E**) Amino acid substitutions within the C-terminus of SHP3 alter its reporter-inducing activity in a Δ*rgg3-shp3* reporter strain (JCC168). sSHP3-C8: DIIIIVGG; the sequences of synthetic peptides with amino acids substitutions are shown in the panel legend. (**F**) sSHP2-C8 and sSHP3-C8 stimulate induction and cross-induction of luciferase at P*_shp2_* (solid lines; BNL148) or P*_shp3_* (dotted lines; JCC174) in wild-type bacteria. Reverse peptide (sSHP2-C8-rev) was included as a control. (**G**) sSHP3-C8 induces a sustained response from the P*_shp2_* reporter in the wild-type strain (BNL148), while luciferase activity of a Δ*rgg3-shp3* strain (BNL150) wanes. (**H**) Synthetic C8 peptides stimulate modest luciferase activity in Δ*rgg2* strains with integrated P*_shp2_* (solid lines; BNL152) or P*_shp3_* (dotted lines; JCC175) reporters.

The expression of *shp* genes in the Δ*rgg3* mutant, in combination with recent discoveries in *S. thermophilus* and *S. mutans* suggesting that *shp* genes encode immature signaling peptides [19], [21], [52], led us to test whether the encoded peptides function as signaling molecules. Since the Δ*rgg3* mutant effectively expresses *shp2* and *shp3* to high levels, as indicated by corresponding reporter strains (Figure 2A and 2B), we focused our investigation on SHP function using this genetic background. Surprisingly, luciferase activity was absent in Δ*rgg3*-*shp3* strains (BNL150 and JCC168, Figure 3A); however, activity could be restored at either promoter when *shp3* was provided from a plasmid (pSHP3). Together, these data suggest that simply removing Rgg3 is not sufficient for reporter induction; instead, activating factors, including SHP3, are required for robust induction. These data also indicate that any polar effects attributable to the construction of the Δ*rgg3*-*shp3* mutant (i.e., disruption of transcription of the downstream biosynthetic operon) do not interfere with SHP3-dependent regulation.

Since microbial cell-to-cell communication systems typically rely on secreted signaling molecules, cell-free culture supernatants from the mutant strains were tested to determine if the reporter-inducing activity was extracellular. Conditioned medium from Δ*rgg3* (JCC131), but not wild type (NZ131) or Δ*rgg3*-*shp3* (JCC132), induced P*_shp3_* luciferase expression in a Δ*rgg3*-*shp3* reporter strain (JCC159), confirming the presence of the activating signal in the extracellular milieu of *shp3*-expressing cells (Figure 3B). In combination with the *shp3* ectopic expression results described above, these data strongly support the role of SHP3, and possibly SHP2, as secreted activating signals.

To begin to address what is required for a functional pheromone signal, a series of truncation mutants was derived from the *shp3* expression plasmid, pSHP3. These plasmids were subsequently electroporated into the Δ*rgg3*-*shp3* mutant (JCC168), and the resulting strains were analyzed for their ability to produce light. Expression of SHP3 residues 15 to 23 (pSHP3_15-23_) resulted in high light production, indicating that the C-terminal portion of SHP3 was sufficient for P*_shp3_*-*luxAB* induction (Figure 3C). Strains containing plasmids encoding versions of SHP3 with deletions of the C-terminal five, three, and final amino acid(s) (pSHP3_1-18_, pSHP3_1-20_, pSHP3_1-22_) did not produce light, demonstrating that an intact C-terminus is also necessary for reporter induction. Finally, in contrast to pSHP3_15-23_, the deletion of 16 amino acids from the N-terminus resulted in the loss of luciferase activity (pSHP3_17-23_). From these data we conclude that P*_shp3_*-inducing activity is located within the last eight or nine amino acids of SHP3. This finding is consistent with recent literature reporting that C-terminal regions of Rgg-associated signaling peptides contain the domain necessary to impart cellular responses [19], [21].

As a complementary approach to the experiments described above, we tested several synthetic versions of SHP3 in the luciferase reporter assay. Synthetic full-length SHP3 (sSHP3), encompassing all 23 amino acids, but not a synthetic peptide with the reversed SHP3 sequence (sSHP3-rev), was able to induce P*_shp3_* in the Δ*rgg3*-*shp3* reporter strain JCC168 (Figure 3D). Luciferase induction was also observed using a synthetic peptide containing the C-terminal eight amino acids (sSHP3-C8). At molar concentrations identical to those used for sSHP3, sSHP3-C8 elicited more than 10-fold greater reporter induction, suggesting that the truncated peptide has higher specific activity. The sSHP3-C8 peptide, a Group I SHP [52], consists of an acidic amino acid followed by a series of hydrophobic amino acids (DIIIIVGG), leading us to wonder if the aspartic acid residue was important for its function. Peptides in which this residue was not included (-IIIIVGG), was substituted with a different acidic residue (EIIIIVGG), or was replaced with the amide-bearing derivative (NIIIIVGG), were tested in our luciferase reporter assay (Figure 3E). Substitution with glutamate retained full activity, but substitution with asparagine decreased activity, and the single residue deletion led to the complete loss of luciferase activity. To test our previous observations that the C-terminal glycine is also important for peptide activity (pJC212 in Figure 3C), we tested DIIIIVG-, and found that its absence also resulted in significantly decreased light activity. Together, these findings indicate that both charge and size of the mature peptide contribute to the ability of SHP3 to affect gene regulation.

Our earlier observation that *shp3* is required for induction at P*_shp2_* (Figure 3A), in combination with the fact that SHP3 and SHP2 amino acid sequences are highly similar, with C-termini identical at seven of eight amino acid positions (Figure 1B), led us to test reporter expression in response to each C8 peptide. Both peptides were equally capable of inducing sustained light production at each promoter in the wild-type genetic background (strains BNL148 and JCC174) while a reverse peptide sSHP2-C8-rev had no effect (Figure 3F). Interestingly, when the experiment was performed using a Δ*rgg3*-*shp3* P*_shp2_* reporter strain (BNL150), we observed initial induction levels comparable to wild-type (BNL148), but reporter activity decreased over time (Figure 3G); similar results were obtained using P*_shp3_* reporter strains (data not shown). This implies that *shp3* autoinduction is required to sustain P*_shp_* expression, and that although *shp2* is induced in response to exogenous peptide, it does not fully compensate for the absence of *shp3*; thus, while both SHPs induce reporter expression, either their activities within the cell are not identical or the dosage effect of having two copies of pheromone is required for lasting autoinduction to occur.

In support of the hypothesis that SHP2 and SHP3 possess distinct activities, a different pattern of reporter induction was observed when C8 peptide was fed to Δ*rgg2* reporter strains. The data presented above suggested that Rgg2 is a positive regulator of transcription at the *shp* promoters (Figure 2); yet in the absence of this regulator we still observed some induction at P*_shp2_* after the addition of synthetic peptide, but no response at P*_shp3_* (Figure 3H). Additionally, sSHP3-C8 had a greater maximal effect than sSHP2-C8 (∼40-fold vs. 7-fold). It is possible that this pattern represents the effect of peptides on Rgg3 repression, in which case SHP3 may have a stronger disruptive effect on Rgg3-DNA interactions than SHP2. Furthermore, it implies that transcription does not strictly require Rgg2, but that the activity or recruitment of RNA polymerase is greater in the presence of this regulator. Taken together, these data strongly support our hypothesis that the SHPs are central to the regulation of Rgg2 and Rgg3 activities at both loci.

### Rgg3 binds DNA and is responsive to sSHP3-C8 *in vitro*

**Figure 4:**
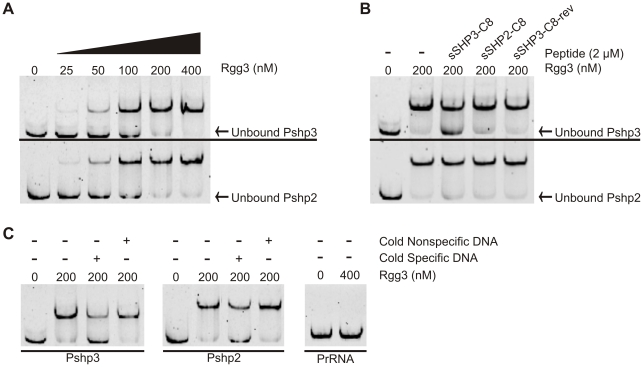
Rgg3 binds to *shp3* and *shp2* promoter regions but can be disrupted by cognate SHPs. EMSA analysis was used to test the ability of recombinant Rgg3 to bind to target promoters and the effect of SHPs on this binding. (A) Rgg3 binds in a concentration-dependent manner to the promoters of both *shp3* (upper panel) and *shp2* (lower panel). (B) Binding to P*_shp3_* by Rgg3 is disrupted by the addition of pure (>95%) sSHP3-C8, and to a lesser extent by pure sSHP2-C8. Both peptides had a smaller effect on disruption of binding to P*_shp2_*. Pure sSHP3-C8-rev was included as a control and did not affect binding to either probe. (C) Binding of P*_shp3_* and P*_shp2_* by Rgg3 is specific and can be disrupted by addition of 5-fold molar excess of unlabeled specific probe but not an equivalent amount of unlabeled rRNA probe (nonspecific). No binding of a negative control rRNA probe (P*_rRNA_*) was detected. All reactions contained 10 nM probe, and 50 nM unlabeled competitor where indicated. Protein concentration is indicated above each lane in all panels.

The conserved helix-turn-helix motif in the N-terminal domain of Rgg2 and Rgg3 proteins strongly suggests a role in DNA binding and is consistent with the properties of other Rgg family members studied to date [46], [53], [54]. To explore the possibility that Rgg2 and Rgg3 are able to bind near promoters of *shp2* and *shp3*, we expressed and purified each protein from *E. coli* with the intent to test DNA binding *in vitro*. Whereas Rgg3 was purified to homogeneity without difficulty, we have been unsuccessful in our attempts to purify soluble Rgg2. Incubation of Rgg3 with fluorescently-labeled P*_shp2_* or P*_shp3_* promoter-region DNA fragments caused a slower migration of the DNA in the gel in a concentration-dependent manner, indicating interaction between Rgg3 and the promoters (Figure 4A). At a molar ratio of 10∶1 Rgg3 to P*_shp3_* or P*_shp2_* DNA, approximately half of the labeled DNA was shifted to the slower migrating band (Figure 4A). Binding of either promoter region was disrupted by five-fold excess unlabeled specific DNA but not nonspecific DNA, and when a fragment of DNA containing a promoter not predicted to be regulated by Rgg3 (P*_rRNA_*) was incubated with the protein, no shift was observed, demonstrating that recognition of DNA sequences by Rgg3 is specific (Figure 4C). Since addition of sSHP3-C8 and sSHP2-C8 peptides to GAS cells resulted in de-repression of the P*_shp2_* reporter (Figure 3H), we asked if these peptides could disrupt Rgg3′s ability to bind DNA *in vitro*. When P*_shp2_* or P*_shp3_* was incubated with Rgg3 in the presence of pure (>95%) sSHP3-C8, Rgg3-DNA complexes were disrupted; however, this disruption was noticeably greater at the *shp3* promoter than at the *shp2* promoter (Figure 4B). Incubation with pure sSHP2-C8 only induced a small amount of disruption of Rgg3 binding, despite the high degree of similarity between SHP2-C8 and SHP3-C8 (Figure 4B). These results are consistent with the enhanced ability of SHP3-C8 over SHP2-C8 to induce luciferase activity in the Δ*rgg2* reporter shown in Figure 3H. Incubation with pure sSHP3-C8-rev had no effects on the Rgg3-DNA interaction, indicating that inhibitory effects of sSHP3-C8 are sequence-specific and are not due to generic properties of the peptide. Importantly, these *in vitro* data suggest that peptide disrupts repression by altering the ability of Rgg3 to bind DNA.

### Biosynthesis and importation of SHPs

**Figure 5:**
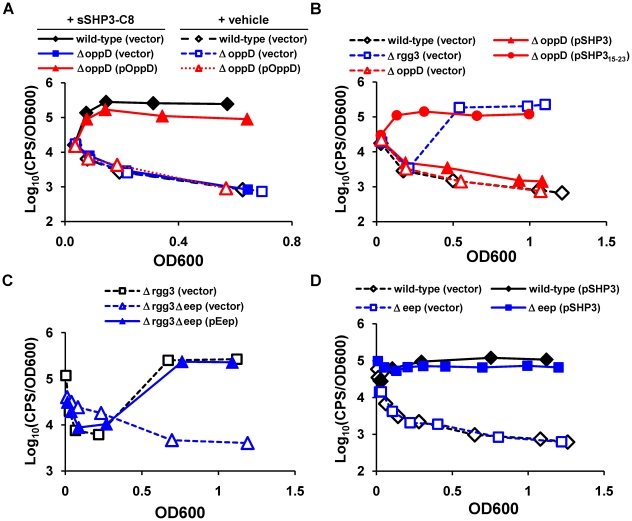
Proteins previously identified in peptide signaling circuits are also important for Rgg-dependent gene regulation. The oligopeptide permease and Eep protease are important for SHP signaling. (A) A Δ*oppD* mutant (JCC163) fails to respond to 50 nM sSHP3-C8 unless an *oppD* complementation vector, pOppD, is present. A wild-type reporter strain carrying empty vector (JCC157 (pLZ12-Sp)) was included as a control. (B) Expression of the C-terminus of SHP3 from a plasmid (pSHP3_15-23_) bypasses the need for the permease in the Δ*oppD* mutant (JCC163). Wild-type (JCC157) and the Δ*rgg3* (JCC158) reporter strains carrying the vector (pLZ12-Sp) alone are included for reference. (C) The Eep metalloprotease contributes to signal production, as determined by measuring luciferase activity of the Δ*rgg3*Δ*eep* mutant (JCC161) expressing *eep* from a multi-copy plasmid (pEep) or carrying empty vector (pLZ12-Sp); Δ*rgg3* (JCC158) was included as a control. (D) Overexpression of *shp3* (pSHP3) restores light induction in the Δ*eep* mutant (JCC160) to near wild-type (JCC157) levels. Data shown are representative of experiments performed at least three times.

The oligopeptide permease, Opp, serves as a promiscuous transporter of peptides used as nutrient sources of amino acids and as a means to recycle cell-wall peptidoglycan [55], [56], but is also a crucial component of peptide-dependent cell signaling in several systems, responsible for transport of Phr peptides of *B. subtilis* [57], PapR of *B. cereus* and *thuringensis* [58], plasmid conjugation pheromones of *Enterococcus faecalis* [59], and competence-inducing peptides ComS and XIP in *S. thermophilus* and *S. mutans* [19], [21]. The GAS genome encodes a single Opp system and a paralogous dipeptide permease peptide transport system (Dpp) [60], [61]. Each locus consists of a substrate binding protein (OppA, DppA), plus structural components comprising a membrane-spanning channel (OppBC/DppBC), and ATPase domains (OppDF/DppDF) that drive translocation of the substrate. We asked if Opp or Dpp are involved in SHP responses in GAS by deleting subunit D of each transporter. The P*_shp3_* reporter was integrated into the genome, and the strains were tested for their ability to produce light. As shown in Figure 5A, P*_shp3_*-*luxAB* expression was inhibited in strains lacking *oppD* (JCC163). Exogenously added synthetic pheromone did not induce expression in the Δ*oppD* mutant unless *oppD* was expressed in *trans* from an episomal plasmid. Similar deletions in the dipeptide permease system did not preclude response to synthetic pheromone, suggesting that Opp is the primary mechanism of signal import (data not shown).

Given the evidence that SHP3 activity is located in the C-terminus of the peptide (Figure 3C and 3D), we next asked whether expression of this portion of the peptide could bypass the requirement for signal import via Opp. As shown in Figure 5B, Δ*oppD* GAS expressing full-length SHP3 (pSHP3) showed only a 2-fold increase in relative luciferase activity compared to cells containing vector only (pLZ12-Sp). In contrast, cells carrying pSHP315-23 exhibited approximately 150-fold more activity than the vector-only control, suggesting that Opp can be bypassed and that the processing of full-length peptide is an important step in pheromone maturation and may occur during the export process or while the pro-peptide is in the extracellular milieu.

Some of what is known about peptide maturation by proteolysis comes from studies on enterococcal plasmid conjugation. For several of these systems, processing of both the pheromones and inhibitor peptides requires a membrane metalloprotease designated Eep [25], [62], [63]. Eep orthologs are important for the processing of a *S. gordonii* pheromone capable of eliciting transfer of an enterococcal conjugative plasmid [64], play a role in lipoprotein processing in *S. uberis* [65], and recently were shown to contribute to processing of SHP-dependent signaling in *S. thermophilus* [52]. The GAS genome contains a single *eep* homologue (*spy49_1620c*), which is 70% identical to the enterococcal protein, containing the HExxH metalloprotease signature and four predicted transmembrane domains [66]. We targeted *spy49_1620c* for deletion in the Δ*rgg3* strain JCC131, and then integrated the P*_shp3_* luciferase reporter, creating JCC161. As shown in Figure 5C, when we monitored light production to determine how Eep affects the Rgg-peptide signaling circuit, the Δ*rgg3*Δ*eep* mutant (JCC161) exhibited greatly decreased (>90%) luciferase activity compared to the Δ*rgg3* mutant (JCC158). Complementation with an episomal copy of *eep* restored high light production normally observed in JCC158, supporting a role for Eep in processing of SHP3 to its functional form. Interestingly, expression of *shp3* from pSHP3 in the *eep* single mutant strain, JCC160, increased light production to levels close to that of wild-type cells carrying the same plasmid, suggesting that Eep is not absolutely required for induction at the P*_shp3_* promoter when SHP3 is abundant, or that other enzymes may compensate when this metalloprotease is absent (Figure 5D).

### Biofilm biogenesis is enhanced by Rgg circuit

**Figure 6:**
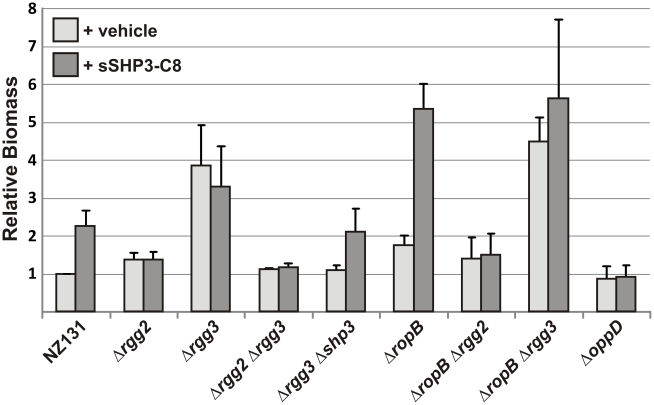
Influence of Rgg regulators on biofilm production. SHPs can enhance biofilm biogenesis via the Rgg2/3 regulatory circuit and counter biofilm disassembly promoted by RopB. Crystal violet staining was performed to measure biofilm biomass of NZ131 derivatives grown 48 hours in 24-well polystyrene plates with (dark gray bars) or without (light gray bars) the addition of 50 nM sSHP3-C8. Levels of biofilm production were normalized to that of untreated NZ131 (wild type). Error bars indicate standard error from a minimum of three independent experiments.

With evidence that the Rgg-SHP system provides GAS with the capability to communicate intercellularly, we wondered what benefits community-wide gene coordination might confer to this organism. As biofilm development is a complex, multicellular process that is commonly regulated by quorum sensing [67], we tested if the Rgg2/3 signaling pathway influenced GAS biofilms. Using a standard crystal violet staining method, we compared biofilm development of wild-type NZ131 with that of *rgg* deletion strains (Figure 6). Although M49 serotypes are poor biofilm producers [68], [69], [70], when *rgg3* was deleted in the M49 strain NZ131 (JCC131), we observed a three-fold increase in crystal violet staining. Consistent with luciferase assay phenotypes reported above, deletion of *rgg2* (JCC137) resulted in very low production of biofilm, similar to that produced by wild-type cells; production remained low even in strains deleted for *rgg3* (BNL145). Recent reports indicate that the secreted cysteine protease SpeB negatively affects production of biofilms in GAS [71], likely because SpeB activity degrades the integrity of proteins contributing to biofilm extracellular matrix. Expression of *speB* requires the positive regulator RopB [45]. In *ropB* mutants incapable of producing SpeB (BNL146), biofilm production was enhanced. When Δ*rgg3* was combined with Δ*ropB* (BNL165), an additive effect on biofilm biogenesis was observed, most likely due to removal of the negative effects on biofilm production contributed by each regulator individually. The increased amount of biofilm observed by deleting *ropB* was eliminated when the *ropB* deletion was combined with Δ*rgg2* (BNL147), implying that if biofilm biogenesis is not initiated, then processes used by bacteria to disrupt biofilms are inconsequential.

Production of biofilms was also enhanced by exogenous addition of sSHP3-C8 (Figure 6). Providing this peptide to wild-type NZ131 cells at a final concentration of 50 nM caused a two-fold increase in biofilm formation, whereas no induction was observed when an equal concentration of reverse peptide was added (data not shown). The sSHP3-C8 also partially complemented the Δ*rgg3-shp3* mutant, but did not further enhance biofilms in the Δ*rgg3* mutant (possibly due to the high amounts of SHP3 already produced by this strain), and could not stimulate biofilms in the Δ*rgg2* or Δ*oppD* mutants. Interestingly, biofilm biomass was increased six-fold when sSHP3-C8 was provided to a Δ*ropB* strain, indicating that cells were more receptive to sSHP3-C8, that biofilms were stabilized in this mutant, or that sSHP3-C8 was stabilized due to lack of SpeB protease. These results indicate a prominent role for the Rgg2/3 pathway and their RopB counterpart in modulating the levels of biofilm produced by GAS.

## Discussion

**Figure 7:**
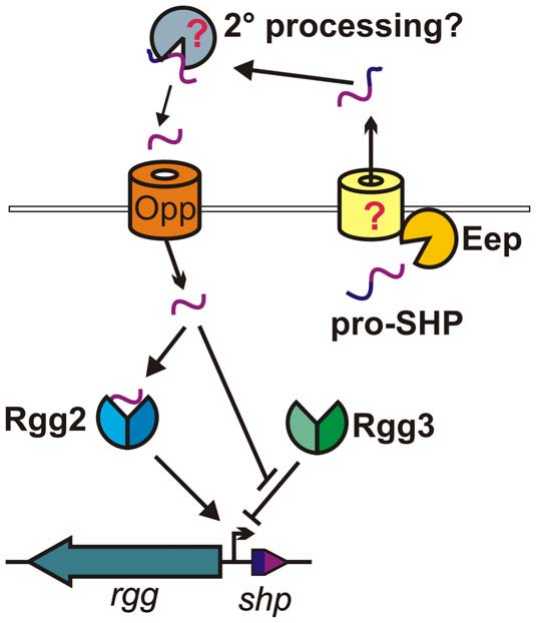
Model for Rgg2/3-SHP2/3 regulation. The pro-SHP peptide is subject to processing by Eep and is secreted by an unknown transporter. Once exported, a secondary processing step may produce active SHP (and, as seen in Figure 5D, process sSHP3). The oligopeptide permease (Opp) imports SHP to the cytoplasm where the pheromone directly engages Rgg proteins. In the absence of SHP, Rgg3 represses transcription of *shp* promoters. De-repression occurs upon Rgg3 binding of either peptide, whereas Rgg2-SHP interactions facilitate transcription activation.

Discoveries over the last forty years have led to a paradigm in which Gram-positive bacteria rely on peptides to coordinate gene expression across a population, recognizing signaling pheromones either extracellularly through sensor histidine kinases, or intracellularly after import via the oligopeptide permease [72]. Pheromone-regulated activities range from expression of virulence factors [73], [74] to sporulation [75] to the exchange of genetic information [76], [77]. However, examples of widely-conserved quorum-sensing systems have largely remained elusive for the pyogenic group of streptococci. Here we present evidence for a new peptide-dependent signaling system in which the opposing activities of two Rgg-family regulators coordinate the expression of neighboring genes in *S. pyogenes*. Our data support a model in which ribosomally-synthesized polypeptides are exported and processed to a mature state, then recognized in the cytoplasm by Rgg regulators after importation of the signal via the oligopeptide permease (Figure 7). Importantly, components of this signaling system are conserved in all sequenced genomes of *S. pyogenes*. This is the first demonstration of Rgg-peptide mediated cell-cell signaling in this species, and the first description of antagonism between any Rgg family members on the basis of small peptides.

Accumulating evidence suggests that Rgg regulators, in combination with cognate peptides, provide a common mechanism for genetic regulation among the streptococci. Functional Rgg-peptide examples now available from *S. thermophilus* (Rgg1358-Shp1358c [20], [52] and ComR-ComS [19]), *S. mutans* (ComR-ComS [21]), and *S. pyogenes* as presented here, substantiate the suggestion that Rgg proteins belong to a family of cytoplasmic regulatory proteins that directly bind peptide ligands. The RNPP family, designated on the basis of its members, which include the Rap phosphatases, NprR and PlcR regulators of *Bacillus* species, and PrgX of *E. faecalis*, consists of quorum-sensing peptide receptors that reside in the cytoplasm [36]. What has distinguished Rgg proteins from the RNPP family thus far is lack of a recognizable tetratricopeptide repeat (TPR) element in Rgg proteins [37] and low sequence similarity between Rggs and RNPP members. Though repeated elements comprising TPR domains are not present in Rgg proteins, structure prediction algorithms align putative alpha-helices of the Rggs to the peptide-binding TPR-domain helices of PlcR and PrgX (PrgX does not contain a *bona fide* TPR domain, but its crystal structure strongly resembles the TPR fold [36]). Studies are underway to determine if the Rgg Conserved Domain does in fact encode a fold similar to the TPR structure; if so, this may expand the possible sequence arrangements that lead to super-helical structures characteristic of TPR domains. If it is determined that Rgg proteins share structural determinants with RNPP members, it would be fitting to amend the group as the RRNPP family [21].

Intercellular signaling among the pyogenic streptococci has been a subject of limited investigation; to date, only one other pathway, named Sil, has been elucidated. This system utilizes a secreted signaling peptide that is detected by a membrane-spanning histidine kinase whose regulation impacts genes encoding a putative bacteriocin biosynthetic pathway. However, conservation of this system is limited to fewer than 20% of GAS isolates [41], [78]. In contrast, all currently sequenced GAS genomes each contain all of the four Rgg paralogs, including Rgg2 and Rgg3, implying that the functions of all four continue to provide evolutionarily-favorable traits.

While we have performed a detailed genetic analysis of the components of a new Rgg-peptide dependent regulatory circuit, relevance of this circuit during the GAS natural life cycle remains unclear. In recent years attention has been given to the ability of GAS to form biofilms, with emphasis placed on their importance during infection and relevance in antibiotic treatment failure [68], [71], [79]. Surprisingly, little is understood about how biofilm communities interact with the host or how these bacteria control this lifestyle. Our results indicate that the Rgg2/3 signaling pathway substantially influences the ability of GAS NZ131 to form biofilms *in vitro*. In a pattern that directly parallels P*_shp_*-luciferase reporters, Rgg2 and Rgg3 play activating and repressing roles, respectively, in generation of biofilm biomass. Whether these regulators directly control genes involved in biofilm development or instead affect downstream regulators remains to be seen. Particularly interesting are the opposing roles that Rgg2/3 and RopB (Rgg1) pathways play in biofilm stasis. The biomass of biofilms was increased after induction of the Rgg2/3 pathway following SHP-dependent stimulation. In contrast, the proteinaceous extracellular matrix of GAS biofilms was disrupted by expression of the SpeB cysteine protease, which is directly activated by RopB [71]. In the absence of *ropB,* biofilm induction by SHPs was greatly enhanced, possibly because decreased protease activity improves peptide-dependent signaling or because other RopB targets counteract a biofilm-promoting lifestyle. Taken together, the opposing roles suggest that these Rgg proteins may provide a switch between promoting and disassembling biofilms. Whereas Rgg2/3 recognize SHPs for activation, no specific signal has been identified that affects RopB. Curiously, RopB requires a cell-density-dependent factor to induce *speB* [46], and we predict another quorum-sensing ligand (possibly another peptide) may be required, which would be consistent with the newly identified function of Rgg proteins as peptide signal receptors.

Other major targets of the Rgg2/3-SHP signaling pathway are the genes located proximally to each Rgg gene. Thus far, homologues of the unusual cluster of genes comprising a putative biosynthetic operon adjacent to *rgg3* (*spy49_0450-0460*) are present only within *Streptococcus pseudoporcinus*, a species recently classified separately from *S. porcinus* genitourinary tract isolates [80], and within a small subset of *B. thuringiensis* and *Salinispora tropica* species [81]. Individual genes within the operon have also been identified in a few other whole genome analyses of GAS, including screens for genes affecting colony morphology (*aroE.2*/*spy49_0450* [82]) and virulence in zebrafish (*spy49_0456* and *mefE/spy49_0460* [83], [84]), and this operon was highly induced in a *mtsR* mutant [49], but a clear understanding of the function of the operon is still lacking. However, it does not appear these genes are involved in biofilm biogenesis, as deletions constructed in the operon did not appreciably affect biofilm production (data not shown). The region including and approximately 1.6 KB downstream of *shp2* was also found to contain transcripts highly responsive to SHP-induction (data not shown). This region of the chromosome is highly conserved (nearly identical nucleic acid sequences) among sequenced GAS genomes, but contains very little recognizable coding sequence. Further downstream lie genes encoding putative bacteriocin peptides and the site at which the *sil* locus is found in isolates that contain this quorum-sensing circuitry [41]. Additional investigation is needed to probe these poorly-studied regions and to identify other targets of regulation and will possibly offer more clues to the function of the Rgg2/3 pathway in the natural life cycle of GAS.

Although the mechanisms by which Rgg2 and Rgg3 regulate gene expression are antagonistic (Rgg3 represses and Rgg2 activates), their response to peptides result in the same inducing effect on P*_shp2_* and P*_shp3_* promoters. Only two other bacterial transcription factors that directly bind peptide pheromones have been studied in detail, and the mechanisms by which they respond to pheromones differ significantly. In the absence of cCF10 pheromone, PrgX exists in a tetrameric state (dimer of dimers) that enhances binding affinity of PrgX for DNA and generates a DNA loop, hindering RNA polymerase access to the P_Q_ promoter. Binding of the pheromone breaks the PrgX dimer interface and disrupts DNA looping, thus de-repressing transcription [85]. In contrast, PlcR is an activator; binding of the PapR pheromone results in a conformation that enhances the interface between HTH domains and target DNA and promotes transcription [36]. The mechanisms by which Rgg2 and Rgg3 each respond to pheromones to control the P*_shp2_* and P*_shp3_* promoters may follow one of these examples, or a new means for pheromone-dependent structural change may emerge. EMSA analysis presented herein supports the latter in the case of Rgg3, as peptide appears to induce dissociation of Rgg3 from target promoters. Especially intriguing is the ability of these highly similar proteins to perform opposing functions. The HTH domains share 72% identity (94% similarity); however, the lower similarity between the putative peptide-binding domains (50% identity, 71% similarity) suggests that this domain may determine the fundamental differences in the regulators' interactions with peptide ligands, RNA polymerase, or, possibly, each other.

SHP2 and SHP3 peptides also share highly similar sequences. Genetic examination of *shp3* indicates the C-terminal eight or nine amino acids encode the minimal required region for reporter induction. The terminal nine residues of each SHP differ by only one hydrophobic amino acid, raising many questions about the peptides' specificity and redundancy in signaling. Surprisingly, differences were seen in Rgg3-DNA interactions in response to the two synthetic peptides. The enhanced ability of sSHP3-C8 to disrupt Rgg3-DNA interaction compared with sSHP2-C8, and the observation that Rgg3 disengages P*_shp3_* at lower concentrations of sSHP3-C8 compared with P*_shp2_*, may reflect ligand specificity and possibly a sequential response in SHP signaling. Why an ordered response matters is difficult to predict without understanding the full repertoire of Rgg-controlled genes, but these observations suggest that the cell is capable of responding to each signal differently and may be able to distinguish peptide concentrations.

While an immediate, robust response was observed upon adding sSHP2-C8 or sSHP3-C8 to reporter strains, precisely how the SHPs are processed and exported out of the cell remains unknown. These hydrophobic molecules might be expected to associate with the cell membrane, but we have shown that cell-free supernatants conditioned by the Δ*rgg3* mutant contain an inducing signal, suggesting active export beyond the cell membrane. In addition, our ability to induce high light production in an *opp*-deficient mutant by expressing the C-terminal nine amino acids (bypassing export/import) argues against posttranslational transport-coupled modification as a prerequisite for peptide function. Our data also demonstrate a role for the site 2 protease (S2P) family member, Eep, in pheromone processing. Much of the characterization of this zinc metalloprotease has taken place in the context of peptide signaling in enterococcal plasmid conjugation. Within this system, evidence suggests that membrane-bound Eep acts directly on the substrate after its translation, recognizing amino acids N-terminal to the active peptide [63]; although the GAS Eep is clearly important in the Rgg-SHP regulatory circuit, we have not yet determined whether it functions identically. Interestingly, the production of at least one enterococcal sex pheromone does not require *eep* [25], raising the possibility that an alternate processing machinery may exist.

Finally, it is intriguing to point out that nearly identical orthologs of *rgg2*/*shp2* are found in *S. agalactiae* and *S. dysgalactiae,* and nearly identical orthologs of *rgg3*/*shp3* are located in *S. pneumoniae, S. thermophilus,* and *Staphylococcus pseudointermedius*. It is tempting to speculate that these signaling pathways provide useful quorum-sensing functions for each species, and that, in the case of *S. pneumoniae* and S. *dysgalactiae* which share the nasopharynx with *S. pyogenes*, interspecies signaling may occur. However, the genetic neighborhoods surrounding *rgg* orthologs in these related species have not been conserved. It is plausible that each SHP-detecting species responds differently to the pheromones, using information contained in this signal to guard itself from competing organisms, or, conceivably, to participate in a multispecies consortium that defines the local ecosystem.

## Methods

### Bacterial strains and media

*S. pyogenes* was routinely grown in Todd-Hewitt (TH; BD Biosciences) supplemented with 0.2% yeast extract (Y; Amresco) or in an improved chemically-defined medium (CDM) based on a previously described recipe [21], [51]. Briefly, we found that the preparation of this medium could be streamlined by combining components into stock solutions that could be stored at −20°C or room temperature (as described in Protocol S1 in Supporting Information). Furthermore, we found that L-asparagine, absent from the original recipe, added to a final concentration of 100 mg L^−1^ supported robust growth of the bacteria. Cultures were stored at −80°C in THY supplemented with 20% glycerol. When appropriate, antibiotics were used at the following concentrations: chloramphenicol (cm, 3 µg mL^−1^), erythromycin (erm, 0.5 µg mL^−1^), spectinomycin (spec, 100 µg mL^−1^). *E. coli* cloning strains DH10β (Invitrogen), BH10c [86], and XL-10 Gold (Stratagene) were maintained in Luria broth (LB) or on Luria agar with the following antibiotics used at the indicated concentrations: cm (10 µg mL^−1^), erm (500 µg mL^−1^), spec (100 µg mL^−1^). *E. coli* expression strain C41(DE3) [87] was maintained on ampicillin (amp) at 100 µg mL^−1^.

### Construction of mutant strains

All *S. pyogenes* strains used in this study were derived from the serotype M49 strain NZ131 [48] and are described in Table 1. Plasmids and primers used are described in Table 2, and Table S1 in the Supporting Information, respectively. To delete *rgg3* (*spy49_0449c*) or *rgg3-shp3* together, a 3008 bp fragment containing *rgg3* and surrounding upstream and downstream regions was amplified with primers spy0533_S2 and spy0533_AS2 and cloned into the *Not*I site of pFED760 to create pLA101. Inverse PCR followed by digestion with *Pac*I allowed replacement of *rgg3* (pJC175, primers del_spy0533_S3 and del_spy0533_AS3) or *rgg3-shp3* (pJC178, primers del_spy0533_S3 and JC137) with a chloramphenicol acetyltransferase (*cat*) cassette (primers cat_S2 and cat_A2; [88]). Vectors to delete *eep* (pJC183), *rgg2* (pJC186), *oppD* (pJC191), or *ropB* (pBL112) were constructed slightly differently. For these, 0.5 to 1.5 kb regions flanking the gene to be deleted were amplified by PCR (pJC183, primers JC141 to JC144; pJC186, primers JC149, JC153 to JC155; pJC191, primers JC162 to JC165; and pBL112, primers BL45 to BL48), purified, then fused together in a second PCR reaction using the outside primers. This fusion product was cloned into pFED760 or its chloramphenicol-resistant derivative, pJC159, to create the deletion vector. Deletion vectors were electroporated into NZ131, and a two-step temperature-dependent selection process was used to isolate mutants of interest [89]. Briefly, cells containing each deletion construct were grown at the permissive temperature, then shifted to 37°C and plated on the appropriate antibiotic to select for bacteria in which the plasmid had integrated at one of the flanking regions. After confirmation of plasmid integration by PCR, cells were grown for approximately 50 generations at the permissive temperature to allow the plasmid to recombine out of the chromosome, and loss of antibiotic resistance was used to identify the desired mutants. Genotypes were confirmed by PCR. This process was repeated to construct double mutants.

**Table 2:** Plasmids used in this study.

Plasmid	Description	Source
p7INT	Shuttle-suicide vector that integrates at streptococcal bacteriophage T12 *attB* site; Erm^R^	[50]
pBL111	DNA fragment containing *shp2* promoter region (500 bp) fused to *luxAB* by PCR and cloned into p7INT	This study
pBL112	pFED760 with DNA fragments flanking *ropB* fused by PCR to created unmarked deletion	This study
pCA102	pET14b-based expression vector with *rgg3* cloned downstream of His-6 and SUMO tags; Amp^R^	[92]; this study
pCN58	Shuttle plasmid containing promoterless *Vibrio fischeri luxAB* genes; Amp^R^, Erm^R^	[90]
pEep	pLZ12-Sp with *eep* cloned under synthetic promoter from pEVP3	This study
pEVP3	Plasmid encoding synthetic promoter and *cat* chloramphenicol resistance cassette; Cm^R^	[88]
pFED760	Shuttle vector pGh9-ISS1 deleted for ISS1 element; temperature-sensitive, Erm^R^	[21], [95]
pJC159	pFED760 with *ermB* replaced by *cat*; Cm^R^	This study
pJC175	pLA101 with *rgg3* replaced by *cat* cassette	This study
pJC178	pLA101 with *rgg3-shp3* replaced by *cat* cassette	This study
pJC183	pFED760 with DNA fragments flanking *eep* fused by PCR to create unmarked deletion	This study
pJC186	pJC159 with DNA fragments flanking *rgg2* fused by PCR to create unmarked deletion	This study
pJC187	DNA fragment containing *shp3-aroE.2* promoter region (524 bp) fused to *luxAB* by PCR and cloned into p7INT	This study
pJC191	pJC159 with DNA fragments flanking *oppD* fused by PCR to create unmarked deletion	This study
pJC205	pJC187 with *shp3* start codon mutated to GGG	This study
pLA101	Fragment encompassing *rgg3* and flanking DNA cloned into pFED760	This study
pLZ12-Sp	Shuttle vector encoding spectinomycin resistance; pWV01 origin; Sp^R^	[91]
pOppD	pLZ12-Sp with *oppD* cloned under synthetic promoter from pEVP3	This study
pRgg2	pLZ12-Sp with *rgg2* cloned under its native promoter	This study
pRgg3	pLZ12-Sp with *rgg3* cloned under its native promoter	This study
pSHP3	pLZ12-Sp with *shp3* cloned under its native promoter	This study
pSHP3_1-18_	pSHP3 with *shp3* C-terminal five amino acids deleted by inverse PCR	This study
pSHP3_1-20_	pSHP3 with *shp3* C-terminal three amino acids deleted by inverse PCR	This study
pSHP3_1-22_	pSHP3 with *shp3* C-terminal glycine deleted by inverse PCR	This study
pSHP3_15-23_	pSHP3 with *shp3* N-terminal 14 amino acids deleted by inverse PCR	This study
pSHP3_17-23_	pSHP3 with *shp3* N-terminal 16 amino acids deleted by inverse PCR; methionine added to peptide sequence to allow translation	This study

Amp, ampicillin; Sp, spectinomycin.

### Construction of *luxAB* reporters

To monitor the expression of *shp2*, 500 bp directly upstream of the open reading frame were amplified using primers BL43/BL44, and *Vibrio fischeri luxAB* genes were amplified from pCN58 [90] with primers BL26/BL27. The promoter product was fused to *luxAB* in a second PCR reaction using BL43/27, and this product was inserted into *Eco*RI and *Bam*HI sites of p7INT [50] to create pBL111. The P*_shp3_* reporter, pJC187, was constructed similarly using primers JC147/158 (524 bp upstream of *aroE.2*) and JC156/JC157 (*luxAB*), except that the promoter-*luxAB* cassette was inserted into p7INT in the opposite orientation. p7INT encodes the T12 bacteriophage integrase and attachment site, *attP*, as well as the *ermB* erythromycin resistance determinant, and lacks a Gram-positive origin of replication. Electroporation of reporter plasmid into *S. pyogenes* with subsequent selection on erythromycin yields transformants in which the plasmid is integrated in single copy at a *tmRNA* gene; insertion at this site was confirmed by PCR for each strain. To create a reporter in which the start codon of *shp3* was mutated to GGG (pJC205), inverse PCR using primers JC139/140 was performed using pJC187 as the starting template. Plasmids containing the desired mutation were identified by sequencing.

### Construction of complementation and truncation plasmids

All complementation plasmids were derived by cloning PCR fragments into pLZ12-Sp [91]. DNA fragments for complementation vectors pRgg2, pRgg3, and pSHP3, including native promoters, were amplified with primers JC174/176, JC131/175, and JC236/237, respectively. PCR products encoding *oppD* (primers JC209/210) and *eep* (primers JC234/235) were fused to a synthetic promoter derived from pEVP3 (primers JC113/208 [88]) by PCR before being inserted into pLZ12-Sp.

SHP3 truncation plasmids were derived from pSHP3 using inverse PCR. Primers with 5′ end restriction sites were designed to anneal to the pSHP3 template excluding nucleotides of interest: pSHP3_1-18_, primers JC181/182; pSHP3_1-20_, primers JC181/227; pSHP3_1-22_, primers JC229/233; pSHP3_15-23_, primers JC200/230; pSHP3_17-23_, primers JC200/231. Products from these PCR reactions were digested with the appropriate restriction enzyme, *Dpn*I-treated to remove template DNA, self-ligated, and electroporated into *E. coli*. Plasmids were confirmed by sequencing before electroporation into *S. pyogenes* strains.

### Luciferase assay

For luciferase assays, isolated colonies from the strains of interest were picked from a freshly streaked plate, inoculated into THY broth and grown overnight at 30°C. In the morning, cells were diluted 100-fold into CDM and incubated at 37°C. At each time point, 50 µL of each culture was removed to an opaque 96-well plate, samples were exposed to decyl aldehyde (Sigma) fumes for one minute, and counts per second (CPS) were quantified using a Wallac 1450 microbeta scintillation counter; the optical density (OD) at 600 nm was also measured at each time point. Relative light units (RLU) were calculated by normalizing CPS to OD. For the conditioned media experiment, strains of interest were grown in CDM to an OD of 0.3 to 0.5, cells were spun down, and supernatants were passed through a 0.22 µm filter; log-phase Δ*rgg3-shp3* reporter cells (JCC159) were diluted 1∶12 into conditioned supernatants, and light activity and OD were monitored as described above. For experiments with synthetic peptides, reporter strains were grown to an OD of 0.3 to 0.5 and then diluted into fresh CDM containing the peptide of interest, and light activity and OD were monitored as described above.

### Synthetic peptides

Synthetic peptides were purchased from Neo-Peptide (Cambridge, MA). Purities of crude preparations used in luciferase assays ranged from 31% to 50%. 95% pure preparations were used for electrophoretic mobility shift assays (EMSA) as described below. All peptides were reconstituted as 2 mM stocks in DMSO and stored in aliquots at -80°C. Subsequent dilutions for working stocks (50 µM) were made in DMSO and stored at −20°C.

### Purification of recombinant Rgg3

The NZ131 *rgg3* gene (*Spy 49_0449c*) was amplified using gene-specific primers (rgg3_fwd_NdeI/rgg3_rev_BamHI) and cloned into a modified pET expression vector bearing N-terminal His-6 and SUMO tags [92]; the resulting vector, pCA102, was electroporated into *E. coli* C41(DE3) before each purification. Expression of *His6-sumo-rgg3* was induced at an OD600 of 0.8 with 0.5 mM IPTG for six hours at 30°C. Cells were pelleted and suspended in Buffer A (20 mM sodium phosphate buffer, pH 7.5; 1.5 mM MgCl_2_, 280 mM NaCl, 20 mM imidazole, 10 mM β-mercaptoethanol) with 0.1 mg mL^−1^ lysozyme, 0.01 mg mL^−1^ DNaseI (Sigma) and Complete EDTA-free protease inhibitor (Roche). Cells were disrupted by sonication on ice, and cellular debris was removed by centrifugation at 45,000 x *g* for 20 min at 4°C. His6-SUMO-Rgg3 was adsorbed to a HisTrap-HP Nickel column (GE Biosciences), then washed with 50 mM imidazole and eluted with 300 mM imidazole. The eluate was immediately exchanged into fresh Buffer A using a Zeba spin desalting column, 7K MWCO (Thermo Scientific). Protein concentration was estimated by measuring the OD at 280 nm. The tagged protein was treated with SUMO protease (a gift of Arnon Lavie lab, [92]) for two hours at room temperature, and re-applied to a nickel column to separate His6-SUMO from Rgg3. Pure Rgg3 was obtained in the flow-through and was concentrated using an Amicon Ultracel 10K centrifuge concentrator (Millipore). Glycerol was added to pure protein to a final concentration of 20%, and aliquots were flash-frozen in a dry ice-ethanol bath and stored at −80°C.

### Electrophoretic mobility shift assays (EMSAs)

All DNA fragments for EMSAs were amplified from chromosomal NZ131 DNA by PCR using primers described in Table S1, with select primers containing 5′-FAM fluorescent tags (Integrated DNA Technologies). The following probes were amplified using primers indicated in parentheses: P*_shp3_* (BL49/JC131), P*_shp2_* (BL50/BL52), and P*_rRNA_* (BL35/BL37). Specific unlabeled competitor DNA for P*_shp3_* and P*_shp2_* were amplified using primer pairs JC131/JC132 and BL51/BL52, respectively. Nonspecific unlabeled competitor P*_rRNA_* DNA was amplified using primers BL35 and BL36. For EMSAs, varying concentrations of recombinant Rgg3 were incubated in 20 µL reactions containing 20 mM HEPES, pH 7.9; 100 mM KCl; 12.5 mM MgCl_2_; 0.2 mM EDTA, pH 8.0; 0.5 mM dithiothreitol; 50 µg mL^−1^ salmon sperm DNA; 0.001 U µL^−1^ poly(dI•dC); 100 µg mL^−1^ bovine serum albumin; 0.5 mM CaCl_2_; and 12% (v/v) glycerol. Following the addition of 10 nM probe, the reactions were incubated at room temperature for 30 minutes. If competitor DNA was included in the reaction, it was added simultaneously with the labeled probe to a final concentration of 50 nM. Any peptide included in the reaction was added to a final concentration of 2 µM 20 min after the addition of probe, and all reactions lacking peptide received an equivalent amount of DMSO vehicle. Sample aliquots of 8 µL were separated on 5% native polyacrylamide gels buffered with 20 mM potassium phosphate, pH 7.5, for 60 min at 100 V at 4°C. All gel shifts were detected by fluorescence imaging using a *Typhoon PhosphorImager* (GE Life Sciences).

### Biofilm assays

Assays were performed as described by Manetti *et al* [93], with slight modifications. Briefly, each strain was grown overnight in THY at 30°C, diluted 1∶10 into fresh CDM, and 0.5 mL volumes were aliquoted to three separate wells of a 24-well polystyrene plate. When indicated, synthetic peptide was added to a final concentration of 50 nM. Plates where incubated at 37°C with 5% CO_2_ for 24 hours, then wells were washed with PBS and fresh CDM (containing peptide, if appropriate) was added. After an additional 24 hours of growth, each well was washed three times with PBS and stained with 0.2% crystal violet for 10 min. Excess crystal violet was aspirated off, and biofilms were washed three times with PBS. The biomass was then suspended in 1% SDS, and the absorbance at 540 nm was measured. Each strain was tested a minimum of three independent times on separate days, and each trial included NZ131 as the reference strain.

## Supporting Information

Protocol S1 Preparation of a chemically-defined medium. The protocol describes the preparation and storage of stock reagents and an improved and streamlined recipe for preparing a chemically-defined medium useful for growing streptococci, based on the recipe described by van de Rijn [51].
(XLS)

Table S1 Primers used in this study.
(DOC)
