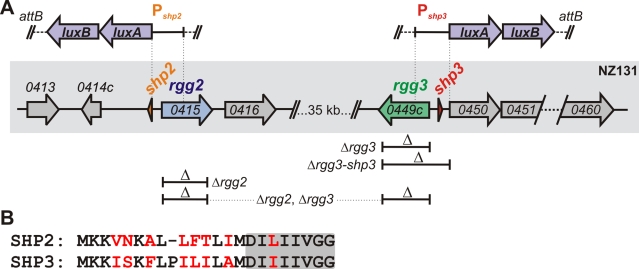# Correction: Two Group A Streptococcal Peptide Pheromones Act through Opposing Rgg Regulators to Control Biofilm Development

**DOI:** 10.1371/annotation/a41fff48-2a84-4cb8-b27c-afd14bcd40f0

**Published:** 2011-10-10

**Authors:** Jennifer C. Chang, Breah LaSarre, Juan C. Jimenez, Chaitanya Aggarwal, Michael J. Federle

In the original Figure 1B, sequences following the SHP2 and SHP3 labels were mistakenly interchanged. The correct sequence for SHP2 is MKKVNKALLFTLIMDILIIVGG, and for SHP3 is MKKISKFLPILILAMDIIIIVGG. Please see the corrected Figure 1 here:

**Figure:**