# Supplementary material for: Two Group A Streptococcal Peptide Pheromones Act through Opposing Rgg Regulators to Control Biofilm Development
Source: PLoS Pathog. 2011 Aug 4;7(8):e1002190. doi: 10.1371/journal.ppat.1002190 (PMC3150281; doi:10.1371/journal.ppat.1002190)
Supplement: Table S1 — Primers used in this study. (DOC) [file ppat.1002190.s002.doc]

**Table S1. Primers used in this study**

| Purpose | Primer name | Sequence | Description |
| --- | --- | --- | --- |
| pBL111 | BL26 | ATGAAGTTTGGAAATATTTGTTTTTCGTATCAACCA | S primer for *luxA* |
|  | BL27 | GCGTGGAATTCGCCTTTAATTTTATTATGGT | AS primer for *luxB*; *Eco*RI |
|  | BL43 | GCGTGGGATCCCCACTTTAAATAAATAATCTGTGAG | S primer for *shp2* promoter; *Bam*HI |
|  | BL44 | ATTTCCAAACTTCATGACTGTCTCCTTTCTGATTTTCTATTTTGT | AS primer for *shp2* promoter; overlap with *luxA* underlined |
| pBL112 | BL45 | GCGTGCTGCAGGACAATTAGCCTTTTAGTTACTATTGTGGC | S primer for *ropB* UFR; *Pst*I |
|  | BL46 | atggatcgttttgcatttaatcgaaatgttTTTTGAATGCCTAAT | AS primer for *ropB* UFR; overlap with DFR underlined |
|  | BL47 | aacatttcgattaaatgcaaaacgatccatATGTTTCAAACC | S primer for *ropB* DFR; overlap with UFR underlined |
|  | BL48 | GCGTGCTGCAGGGTTAGAAGCAAATCGTGATGGCTATCTTTA | AS primer for *ropB* DFR; *Pst*I |
| pCA102 | rgg3 fwd NdeI | GCGTGcatatgATGAAATCAAAACTTGGTGCCACAC | S primer for *rgg3* expression vector; *Nde*I |
|  | rgg3 rev BamHI | GCGTGggatccTTAATCTTTGTTAACTAATTTATCAAAATGG | AS primer for *rgg3* expression vector; *Bam*HI |
| pEep, pOppD | JC208 | CATGGAATTCATTTGTTTGATTTTTAATGG | S primer for synthetic *cat3T* promoter; *Eco*RI |
|  | JC113 | CATTTGATATGCCTCCTAAA | AS primer for synthetic *cat3T* promoter |
| pEep | JC234 | AGGAGGCATATCAAATGTTAGGAATAATAACC | S primer for *eep*; overlap with synthetic promoter underlined |
|  | JC235 | CATGAGATCTGCAAATCTTAGGGTTGAGG | AS primer for *eep*; *Bgl*II |
| pJC175 | del_Spy0533_S3 | GCGTGTTAATTAATAAGTGAAATCATTCTTTACCTTTTAACG | Inverse PCR primer; *Pac*I |
|  | del_Spy0533_AS3 | GCGTGTTAATTAAATTTTCCCACTTTCCCAACAAAAATCAATTC | Inverse PCR primer; *Pac*I |
|  | cat_S2 | GCGTGTTAATTAAAAGTATCGATAAGCTTGATGAA | *cat* cassette from pEVP3; *Pac*I |
|  | cat_A2 | GCGTGTTAATTAAAAGAAAAAGGAGAAGTCG | *cat* cassette from pEVP3; *Pac*I |
| pJC178 | JC137 | CATGTTAATTAATTATTGTTGGTGGGTAAGTTACTTAGGTG | Inverse PCR primer; *Pac*I |
| pJC183 | JC141 | CATGGCGGCCGCGTCGTTTACCTGAAGATACC | S primer for *eep* UFR; *Not*I |
|  | JC142 | GACCTATCTTGATTACATAGATTTCCTTTCAGTC | AS primer for *eep* UFR; overlap with *eep* DFR underlined |
|  | JC143 | GAAAGGAAATCTATGTAATCAAGATAGGTCATC | S primer for *eep* DFR; overlap with *eep* UFR underlined |
|  | JC144 | CATGCTCGAGCTGCACCAAAGAAAGCACG | AS primer for *eep* DFR; *Xho*I |
| pJC186 | JC149 | CATGGAATTCATGTAGTTGTTGTTAGTTTAGCC | S primer for *rgg2* UFR; *Eco*RI |
|  | JC153 | CTGATAGAAAGCAACTCACATTTTTCCCACTTTCACAAC | AS primer for *rgg2* UFR; overlap with DFR underlined |
|  | JC154 | TGTGAAAGTGGGAAAAATGTGAGTTGCTTTCTATCAGATG | S primer for *rgg2* DFR; overlap with UFR underlined |
|  | JC155 | CATGGAATTCTTTCCTGATGCAATGCCTCC | AS primer for *rgg2* DFR; *Eco*RI |
| pJC187 | JC147 | CATGGAATTCGTGTCGGAAAGTAAACATGC | S primer for *shp3*-*aroE.2* promoter region; *Eco*RI |
|  | JC158 | CAAATATTTCCAAACTTCATTACACAGAGCTAACCATTACC | AS primer for *shp3-aroE.2* promoter; *luxA* overlap underlined |
|  | JC156 | ATGAAGTTTGGAAATATTTGTTTTTCG | S primer for *V. fischeri luxA* |
|  | JC157 | CATGGGATCCTTGCCTTTAATTTTATTATGG | AS primer for *V. fischeri luxB*; *Bam*HI |
| pJC191 | JC162 | CATGCTCGAGCAACTACGGTTATCTGGTTCC | S primer for *oppD* UFR; *Xho*I |
|  | JC163 | CATTTCCTTCCTCCTGTTTAACTCCTATCTATGTGAACG | AS primer for *oppD* UFR; overlap with DFR underlined |
|  | JC164 | CATAGATAGGAGTTAAACAGGAGGAAGGAAATGTCTG | S primer for *oppD* DFR; overlap with UFR underlined |
|  | JC165 | CATGCTCGAGGAACTCCGTTTGAAAGAAGC | AS primer for *oppD* DFR; *Xho*I |
| pJC205 | JC139 | CAATAAATAAAAACTGAAAGGAAGTCCACTTGGGAAGAAAATTTCAAAATTTTTGCCGATTTTA | nucleotide changes underlined |
|  | JC140 | TAAAATCGGCAAAAATTTTGAAATTTTCTTCCCAAGTGGACTTCCTTTCAGTTTTTATTTATTG | nucleotide changes underlined |
| pLA101 | spy0533_S2 | GCGTGGCGGCCGCGAAAAAAAATATT | S primer for *rgg3* region; *Not*I |
|  | spy0533_AS2 | GCGTGGCGGCCGCAAAGCGAAAAAA | AS primer for *rgg3* region; *Not*I |
| pOppD | JC209 | AGGAGGCATATCAAATGACAAAAGAAAATAATGTAATC | S primer for *oppD*; overlap with synthetic promoter underlined |
|  | JC210 | CATGAGATCTTTATTCTAGGTCTTTTACTTCG | AS primer for *oppD*; *Bgl*II |
| pRgg2 | JC236 | CATGGAATTCCATGACTGTCTCCTTTCTG | S primer for *rgg2* complementation; *Eco*RI |
|  | JC237 | CATGAGATCTAAAGGACAGCTAGACCC | AS primer for *rgg2* complementation; *Bgl*II |
| pRgg3 | JC176 | CATGAGATCTATAAGTGGACTTCCTTTCAG | S primer for *rgg3* complementation; *Bgl*II |
|  | JC174 | CATGAGATCTAGAAGCTCAAGAGATGACC | AS primer for *rgg3* complementation; *Bgl*II |
| pSHP3 | JC131 | CATGGAATTCATATTTTCCCACTTTCCC | S primer for *shp3* complementation; *Eco*RI |
|  | JC175 | CATGAGATCTTTAAAATTTCATCCTCCTACG | AS primer for *shp3* complementation; *Bgl*II |
| pSHP3_1-18_, pSHP3_1-20_ | JC181 | CATGTGATCATGGTGGGTAAGTTACTTAGG | Inverse PCR S primer; *Bcl*I |
| pSHP3_1-18_ | JC182 | CATGTGATCAGATAATATCCATTGCTAAAATT | Inverse PCR AS primer; *Bcl*I |
| pSHP3_1-20_ | JC227 | CATGTGATCAAATAATGATAATATCCATTGCTAA | Inverse PCR AS primer; *Bcl*I |
| pSHP3_1-22_ | JC233 | CATGTGATCATAAGTTACTTAGGTGTATACC | Inverse PCR S primer; *Bcl*I |
|  | JC229 | CATGTGATCAACCAACAATAATGATAATATCCA | Inverse PCR AS primer; *Bcl*I |
| pSHP3_15-23_, pSHP3_17-23_ | JC200 | CATGGGATCCACTTCCTTTCAGTTTTTATTTATTG | Inverse PCR AS primer; *Bam*HI |
| pSHP3_15-23_ | JC230 | CATGGGATCCATGGATATTATCATTATTGTTGG | Inverse PCR S primer; *Bam*HI |
| pSHP3_17-23_ | JC231 | CATGGGATCCATGATTATCATTATTGTTGGTGGGTA | Inverse PCR S primer; *Bam*HI |
| EMSA | BL49 | 5' 6-FAM-TACACAGAGCTAACCATTACC | Fluorescently labeled primer for *shp3* promoter region |
|  | JC132 | CATCCGTCTCTCTCCTTTACACAGAGCTAACCATTACC | *shp3* promoter region |
|  | BL50 | 5' 6-FAM-TATTCATCTGCTAAAAAAGAAATGCTAACCTGC | Fluorescently labeled primer for *shp2* promoter region |
|  | BL51 | TATTCATCTGCTAAAAAAGAAATGCTAACCTGC | *shp2* promoter region |
|  | BL52 | TTTCCCCAAAGTGGATTGTTTTTAAGC | *shp2* promoter region |
|  | BL35 | GTGTTTAAAGACCTCTCATGGGCAAAT | *rRNA* promoter region^a^ |
|  | BL36 | CAGGTTTCTCATAGCCTGTCAACTACTTTT | *rRNA* promoter region^a^ |
|  | BL37 | 5' 6-FAM-CAGGTTTCTCATAGCCTGTCAACTACTTTT | Fluorescently labeled primer for *rRNA* promoter region^a^ |

S, sense; AS, antisense; UFR, upstream flanking region; DFR, downstream flanking region; 5’ 6-FAM, 6-carboxyfluorescein at 5’ end.

^a^ LaSarre B, Federle MJ (2011) Regulation and Consequence of Serine Catabolism in *Streptococcus pyogenes*. *J Bacteriol* 193: 2002-2012.
